# Validation of the Comprehensive Feeding Practices Questionnaire (CFPQ) with Portuguese Caregivers of 2-to-8-Year-Olds

**DOI:** 10.3390/children10121924

**Published:** 2023-12-14

**Authors:** Ana F. Santos, Marília Fernandes, Carla Fernandes, Luísa Barros, Manuela Veríssimo

**Affiliations:** 1 William James Center for Research, ISPA-Instituto Universitário, 1149-041 Lisboa, Portugal; afsantos@ispa.pt (A.F.S.); mfernandes@ispa.pt (M.F.); csfernandes@ispa.pt (C.F.); 2 Centro de Investigação em Ciência Psicológica, Faculdade de Psicologia, Universidade de Lisboa, Alameda da Universidade, 1649-013 Lisboa, Portugal; lbarros@psicologia.ulisboa.pt

**Keywords:** parental feeding practices, responsive feeding, factorial structure, validation, children

## Abstract

Recent findings have demonstrated an increase in the prevalence of childhood obesity and overweight in Portugal, urging the need to study modifiable risk factors such as parental feeding practices. The Comprehensive Feeding Practices Questionnaire (CFPQ) is an important self-report measure assessing a broad range of responsive and non-responsive feeding practices. However, the CFPQ has not yet been validated in Portugal. Therefore, the present study aimed to test the validity of this measure with Portuguese parents of 2-to-8-year-old children. A sample of 409 parents completed a Portuguese-adapted version of the CFPQ and the already validated Child Feeding Questionnaire (CFQ). Confirmatory factor analysis (CFA), exploratory factor analysis (EFA), and psychometric analysis were conducted. CFA demonstrated the original 12-factor structure did not fit the sample. EFA identified an eight-factor structure comprising 29 items: Monitoring, Modeling, Environment, Involvement, Emotion Regulation, Restriction for Weight Control, Restriction for Health, and Pressure. Findings suggest that parental feeding practices are sensitive to parents’ background cultures and children’s developmental period.

## 1. Introduction

Obesity has reached epidemic proportions in Europe and is still escalating, posing an increasing health challenge. According to the WHO Regional Office for Europe [1], obesity and overweight affect 7.9% of children under the age of five (4.4 million children) and one in three school-aged children. Portugal is one of the countries in Europe with the highest prevalence of childhood obesity and overweight, with 31.6% and 13.5% of children being overweight and obese, respectively [2,3]. Overweight and obese children tend to remain so in adulthood [4,5,6] and are more likely to develop non-transmissible diseases, such as cardiovascular diseases, diabetes, musculoskeletal disorders, and cancer [7,8,9]. Thus, it is of great interest to investigate modifiable risk factors for obesity in infancy and childhood.

In this sense, a large body of evidence shows that parental feeding practices influence children’s eating behaviors and weight status [10,11,12,13,14]. Children are born with the ability to regulate their energy intake [15,16,17]. Nevertheless, as children age, this ability decreases due to external influences [18,19,20,21,22], namely parental feeding practices [23,24]. Therefore, feeding practices can either support or undermine children’s ability to self-regulate their internal hunger and satiety cues, depending on whether they are responsive or non-responsive to these cues [25]. Responsive feeding practices refer to parents’ capacity to correctly recognize and respond to these cues, thus positively influencing children’s self-regulation of energy intake. On the contrary, non-responsive feeding practices refer to the use of excessive controlling and coercive feeding practices that teach children to eat for reasons unrelated to their appetite and ignore their hunger and satiety cues [23,26,27,28,29,30]. These practices can compromise children’s inborn capacity to regulate their energy intake and contribute to poor diet quality and excessive weight gain [12,26,27,29,31,32,33].

Most studies have focused on non-responsive feeding practices, such as Restriction and Pressure to Eat [34,35,36,37,38,39], with responsive feeding practices being less studied. As such, the Child Feeding Questionnaire (CFQ) [40] has been one of the most widely used instruments to measure parental feeding practices; however, this instrument assesses only three feeding practices: Monitoring, Pressure to Eat, and Restriction (with the two last ones being non-responsive practices). Therefore, researchers have highlighted the importance of including other less controlling and coercive feeding practices, especially those associated with healthy outcomes [33,41]. With the development of the Comprehensive Feeding Practices Questionnaire (CFPQ) [42], the CFQ and other previous measures were expanded. The CFPQ goes beyond controlling feeding practices, including a broader range of feeding practices, namely those that are responsive, such as modeling healthy eating, teaching about nutrition, and encouraging balance and variety [42]. Thus, the CFPQ provides a more comprehensive description of parental feeding practices.

Given the high prevalence of obesity and overweight among Portuguese children [2], it is important to study and understand parental feeding practices. Even so, there is a lack of valid measures for the Portuguese population that assess feeding practices. To our knowledge, only two measures were validated in Portugal—the CFQ [43,44] and the Parental Feeding Style Questionnaire [45]—which focus more on controlling feeding practices, while one—the Children’s Intake Self-Regulation Feeding Practices Scale [46]—was created and developed in Portugal. The CFPQ has been validated in other countries (e.g., [47,48,49,50,51]) but not yet among the Portuguese population, which limits its generalizability and use in other cultures. Since feeding practices can be influenced by ethnicity [52,53], it is necessary to validate measures for different populations.

Thus, the aim of this study is to validate the CFPQ with Portuguese parents of 2-to-8-year-old children using a confirmatory factor analysis and examine its psychometric properties. If the original CFPQ factor structure is not confirmed, an exploratory factor analysis will be conducted to identify an alternative factor structure.

## 2. Materials and Methods

### 2.1. Participants

The initial sample included 505 caregivers, and although none of the variables presented more than 2.2% missing, the final sample only uses completed data (*n* = 409, 80.99% of the cases). Caregivers were mostly mothers (98%) of children between 2 and 8 years (*M* = 49.51 months; *SD* = 13.04). Most children were boys (*n* = 224, 54.8%, and girls, 45.2%), first-born (*n* = 280, 68.5%), and had siblings (*n* = 262, 64.1%). Mothers’ ages ranged between 22 and 50 years (*M* = 35.91; *SD* = 5.36), and fathers between 22 and 59 years (*M* = 37.98; *SD* = 6.17). Mothers’ education levels varied between 8 and 21 years (*M* = 15.99; *SD* = 2.93), and fathers between 5 and 21 years (*M* = 14.33; *SD* = 3.41). Most caregivers were married or cohabiting (86.6%) and worked full-time (79.3% mothers; 93.8% fathers).

### 2.2. Measures

#### 2.2.1. Comprehensive Feeding Practices Questionnaire (CFPQ) [42]

The CFPQ is a self-report instrument containing 49 items measuring parental feeding practices for which parents of 2-to-8-year-old children respond on a 5-point Likert scale, indicating their degree of agreement (1 = disagree to 5 = agree; items 1–13) or their frequency of use a specific feeding approach (1 = never to 5 = always; items 14–49). The items are distributed over 12 subscales; 7 of these subscales reflect more positive or responsive feeding practices: (1) *Monitoring*, i.e., how much caregivers keep track of the child’s intake of unhealthy foods (four items, e.g., “How much do you keep track of the sweets that your child eats?”); (2) *Modeling*, i.e., caregivers actively demonstrating healthy eating and being enthusiastic about it (four items, e.g., “I model healthy eating for the child by eating healthy foods myself”); (3) *Teaching about Nutrition*, i.e., how much the caregivers teach and use didactic techniques to encourage the child’s intake of healthy foods (three items, e.g., “I discuss with my child the nutritional value of foods”); (4) *Encourage Balance and Variety*, i.e., the degree to which caregivers promote healthy and varied food consumption (four items, e.g., “I encourage my child to eat a variety of foods”); (5) *Environment*, i.e., how much caregivers make healthy foods available in the house and provide them at mealtimes (four items, e.g., “Most of the food I keep in the house is healthy”); (6) *Involvement*, i.e., caregivers’ promotion of the child’s involvement in meal planning and food preparation (three items, e.g., “I involve my child in planning family meals”); and (7) *Child Control*, i.e., the degree to which caregivers allow the child to make decisions related to their eating behaviors and control feeding interactions (five items, e.g., “At dinner, do you let the child choose the foods she/he wants from what is served?”). The other five subscales reflect non-responsive feeding practices: (8) *Emotion Regulation*, i.e., caregivers’ use of food to regulate the child’s emotions (three items, e.g., “Do you give the child something to eat or drink if she/he is bored even if you think she/he is not hungry?”); (9) *Food as Reward*, i.e., caregivers’ use of food to control the child’s behavior (three items, e.g., “I offer the child his/her favorite foods in exchange for good behavior”); (10) *Restriction for Weight Control*, i.e., how much caregivers control the child’s intake with the purpose of decreasing or maintaining the child’s weight (eight items, e.g., “I encourage the child to eat less so he/she won’t get fat”); (11) *Restriction for Health*, i.e., how much caregivers control the child’s intake with the purpose of limiting unhealthy foods (four items, e.g., “If I did not guide or regulate the child’s eating, he/she would eat too many junk foods”); and (12) *Pressure*, i.e., the degree to which caregivers encourage the child to eat more food, disregarding the child’s satiety and hunger cues (four items, e.g., “My child should always eat all of the food on his/her plate”). Items 16, 37, and 42 are reverse coded. An already translated and adapted Portuguese version was used [54].

#### 2.2.2. Child Feeding Questionnaire (CFQ) [40]

The CFQ comprises 31 items assessing parental feeding practices, parents’ perceptions of parental and children’s weight status, and their concerns about children’s weight. The CFQ was designed for use with parents of 2-to-11-year-old children. Items are answered on a 5-point Likert scale, ranging from 1 (never/disagree) to 5 (always/agree). For this study, only the three subscales measuring feeding practices were used: (1) *Monitoring* (three items, e.g., “How much do you keep track of the high-fat foods that the child eats?”), (2) *Pressure to Eat* (four items, e.g., “If my child says ‘I’m not hungry’, I try to get him/her to eat anyway”), and (3) *Restriction* (eight items, e.g., “I have to be sure that my child does not eat too many high-fat foods”).

### 2.3. Procedures

This study is part of the research project “ChildObesity—Child obesity risk: the Role of Attachment, child’s Temperament and Self-regulation”, and it was approved by the Ethics Committee of the ISPA—Instituto Universitário (I/038/06/2020).

Participants were recruited through convenience sampling, guaranteeing the confidentiality of data collecting process. Part of the sample was recruited via private and public schools in the Lisbon Metropolitan Area (26.5%). Schools’ boards were contacted to present this study’s goals and procedures. After their authorization, informed consent and questionnaires were sent to caregivers through their children’s teachers and returned in a closed envelope after completion. The other part of the sample (63.5%) was recruited online through social network posts (e.g., Facebook and Instagram). The questionnaires were administered through Qualtrics survey platform. When accessing the hyperlink, participants were informed about the aims of this study and about their rights (e.g., confidentiality, withdrawal by closing the browser window). Participants who provided informed consent by clicking on the “I agree” option were redirected to the questionnaires. Duplicated internet protocol (IP) addresses were checked to assess whether there was more than one response from the same IP [55].

### 2.4. Data Analysis

Data analysis was performed using IBM SPSS Statistics, version 29.0, and Jamovi, version 2.3.28 (*SEM* 0.9.5 package). The psychometric sensitivity of the items was assessed with skewness (*sk*) and kurtosis (*ku*), with absolute values of *sk* smaller than 3 and *ku* smaller than 10 considered adequate [56]. Construct validity was assessed through factorial and subsequent convergent and discriminant validity. The factorial validity was analyzed using a confirmatory factor analysis (CFA). The goodness of fit was provided by the relative chi-square fit index (χ^2^/df), the comparative fit index (CFI), the Tucker–Lewis fit index (TLI), the root mean square error of approximation (RMSEA), and the standardized root mean square residual (SRMR). The model’s fit was considered adequate when CFI and TLI ≥ 0.90–0.95, RMSEA ≤ 0.08–0.06, and SRMR ≤ 0.10–0.08 [57,58]. Items that performed poorly in the CFA (i.e., factor loading < 0.40 and/or negative factor loading or commonalities < 0.30) were analyzed and removed to improve model fit. The reliability of the final model subscales of the final model was examined using Cronbach’s alpha (>0.60) [59]. Measurement invariance was also tested [60]. Configural invariance was tested by constraining the basic factor model to equality across the considered groups. If the model fit reasonably well, configural invariance was achieved. To test for metric invariance, equality constraints on the factor loadings were specifying if ΔCFI ≤ 0.01, ΔRMSEA ≤ 0.015, and ΔSRMR ≤ 0.03 weak was achieved. To test for scalar invariance, intercepts were constrained to equality across groups, and a strong invariance was achieved if ΔCFI ≤ 0.01, ΔRMSEA ≤ 0.015, and ΔSRMR ≤ 0.01. Finally, for strict invariance, residuals were also constrained to equality across groups. Using a smaller sample (*n* = 315), the associations between CFPQ and CFQ were analyzed to test for convergent and discriminant validity.

Since various studies found modified versions of the CFPQ structure, an exploratory factor analysis (EFA) was also performed to identify the appropriate alternative CFPQ structure. Factors were hypothesized to correlate; thus, oblique rotation was used. Items that performed poorly (i.e., factor loading < 0.40 and/or negative factor loading) were analyzed and removed. Items cross-loading ≥ 0.40 on multiple factors were also removed. The Kaiser–Meyer–Olkin measure of sampling adequacy (MSA) was applied and required to be ≥0.60 for the overall instrument and each item. Factors with Cronbach’s α scores lower than 0.60 were removed.

## 3. Results

Almost all items showed adequate psychometric sensitivity (|*Sk*| = 0.05 to 2.42; |*Ku*| = 0.02 to 7.38), except for item 45 (“I often put my child on a diet to control his/her weight” with |*Sk*| = 5.52 and |*Ku*|= 33.01; *M* = 1.09, *SD* = 0.45).

### 3.1. Confirmatory Factor Analysis

The CFA of the original 12-factor structure did not present an acceptable fit for all the considered indexes (CFI = 0.816, TLI = 0.796, RMSE = 0.050, and SRMR = 0.069). To improve the model fit, four items presenting factor loadings lower than 0.40 were removed (items 18 and 45 from *Restriction for Weight Control* subscale, *λ* = 0.21 and *λ* = 0.36, respectively; item 44 from *Modeling*, *λ* = 0.30; and item 12 from *Child Control*, *λ* = 0.36). Although the model fit did show an improvement, it was still not acceptable (CFI = 0.853, TLI = 0.834, RMSEA = 0.047, and SRMR = 0.063). Four items with low communality (<0.30) were gradually removed: item 10 from *Child Control* (*h*^2^ = 0.19 and *λ* = 0.44); item 43 from *Restriction for Health* (*h*^2^ = 0.20 and *λ* = 0.43); item 13 from *Balance and Variety* (*h*^2^ = 0.21 and *λ* = 0.46); item 17 from *Pressure* (*h*^2^ = 0.20 and *λ* = 0.45); items 31 and 42 from *Teaching about Nutrition* (*h*^2^ = 0.21, *λ* = 0.47 and *h*^2^ = 0.21, *λ* = 0.47, respectively); and finally, item 11 from *Child Control* (*h*^2^ = 0.23 and *λ* = 0.49) and residuals of item 34 and 35 were correlated (MI = 83.4). Most of the considered model fit indexes were acceptable (CFI = 0.913, TLI = 0.898, RMSEA = 0.041, and SRMR = 0.054). Internal consistency reliability of the subscales was examined using a 12-factor final model. However, the *Teaching about Nutrition* subscale was reduced to one item and was removed. All the subscales showed acceptable reliability with α ≥ 0.60 (from 0.63 to 0.85), except for encouragement for *Child Control* (α = 0.46) and *Food as Reward* (α = 0.59) (see Table 1).

Using the final 11-factor model with 37 items, measurement invariance across sex was tested. Results suggest configural invariance as the model fits reasonably well (CFI = 0.892, RMSEA = 0.047, and SRMR = 0.065). Strict invariance across groups was achieved; more specifically, loading (ΔCFI = 0.002, ΔRMSEA = 0.000, and ΔSRMR = 0.001), intercepts (ΔCFI = 0.002, ΔRMSEA = 0.001, and ΔSRMR = 0.001), and residual invariance (ΔCFI = 0.007, ΔRMSEA = 0.001, and ΔSRMR = 0.000) were achieved.

### 3.2. Exploratory Factor Analysis

EFA was also explored. In the first EFA, a nine-factor structure was found. The Kaiser–Meyer–Olkin MSA was acceptable for the overall measure and for each item (overall MSA = 0.79; for the items, MSA ranged from 0.64 to 0.89). However, fifteen items performed poorly and were removed (5, 6, 10, 11, 13, 16, 18, 19, 22, 24, 26, 36, 38, 40, 45). In the second EFA, five items performed poorly and were removed (12, 23, 31, 37, 42). In the third EFA, an eight-factor structure was found; items 26 and 37 presented low-factor loadings and were removed. The final EFA presented an eight-factor structure (see Table 2). MSA was adequate for the overall measure and for each item (overall MSA = 0.74; for the items, MSA ranged from 0.63 to 0.84).

### 3.3. Associations between Modified CFPQ Subscales and CFQ Subscales

Relation to subscales from the CFQ was also explored (see Table 3). The majority of the associations found between the modified CFPQ subscales and the CFQ subscales were in expected directions, except for the positive association between CFPQ Modeling and CFQ Restriction (r = 0.25, *p* < 0.001), the negative association between CFPQ Environment and CFQ Monitoring (r = –0.14, *p* < 0.05), and the positive association between CFPQ Restriction for Health and the CFQ Monitoring (r = 0.22, *p* < 0.001).

## 4. Discussion

This study aimed to examine the factor structure proposed by Musher-Eizenman and Holub [42] of the Comprehensive Feeding Practices Questionnaire in a Portuguese sample of caregivers of children between 2 and 8 years old. Confirmatory factor analyses showed the lack of fit of the original 12-factor and 49-item model in this sample, which is in line with previous findings from other validation studies in other cultures (e.g., [47,48,50,51,61,62,63]). These studies proposed different structures for the CFPQ. For example, Melbye et al.’s [64] study with a sample of parents of 10-to-12-year-olds in Norway suggested a 10-factor model, including 42 items. In New Zealand, Haszard et al. [48] identified a five-factor model with 32 items in a sample of parents of 4-to-6-year-olds. Shohaimi et al.’s [51] study with Malaysian mothers of 7-to-9-year-olds identified a 12-factor model with 39 items. The study of Mais et al. [50] with Brazilian parents of 5-to-9-year-olds proposed a six-factor model, including 42 items, which was confirmed in another sample of Brazilian parents of 2-to-5-year-olds [63]. In Jordan, Al-Qerem et al. [47] identified an 11-factor model with 43 items in a sample of mothers of 6-to-12-year-olds. In our study, exploratory factor analysis suggested that the most suitable structure was 29 items distributed over eight factors: Monitoring, Emotion Regulation, Restriction for Weight Control, Modeling, Pressure, Restriction for Health, Environment, and Involvement.

The different CFPQ structures identified over several validation studies could be explained by cultural and social differences but also methodological differences. Indeed, these studies were conducted with parents of children of different group ages, which could influence the final models of the CFPQ. As demonstrated by Saltzman et al. [65], some feeding practices could be more significant to caregivers at different developmental phases. Thus, their use of particular feeding practices may change with children’s growth. In their study, Saltzman et al. [65] analyzed the factor structure of the CFPQ across two time points. At Time 1, when children were, on average, 37 months of age, a seven-factor model presented the most appropriate fit. At Time 2, at 57 months of age, a five-factor model was the most suitable structure. For instance, when children were about 37 months of age, the Emotion Regulation and Food as Reward subscales were found, but not at 57 months. In this sense, these feeding practices could be more appropriate for parents of toddlers, and their use may decrease as children age [48,65]. On the other hand, the Restriction for Health subscale emerged only at Time 2, suggesting that this feeding practice could be more appropriate for parents of preschoolers [65]. Other feeding practices, including Teaching about Nutrition, Encourage Balance and Variety, and Child Control, could be more suitable for parents of school-aged children [47,48,65]. This could explain why, in the present study, these last three feeding practices were not found since most of our sample was composed of 3-to-5-year-olds (86.8%). In this sense, it is unsurprising that we also did not find the Food as Reward subscale, as the proportion of toddlers in our sample was small.

Compared to the original CFPQ 12-factor model [42], in our 8-factor model, most of the items were loaded in their respective factor. The subscales Monitoring, Emotion Regulation, and Pressure had the same composition as the original factor. The subscales Restriction for Weight Control and Restriction for Health lost two (item 18, “I have to be sure that my child does not eat too many high-fat foods”; item 45, “I often put my child on a diet to control his/her weight”) and one item(s) (item 40, “I have to be sure that my child does not eat too much of his/her favorite foods”), respectively.

The Modeling subscale lost one item (item 44, “I model healthy eating for my child by eating healthy foods myself”) that loaded in the Environment subscale. This result was also found in the study of Melbye et al. [64]. As explained by Melbye et al. [64], the other three items in the Modeling subscale appear to reflect a more active form of Modeling (e.g., “I try to eat healthy foods in front of my child…”; “I show my child how much I enjoy eating healthy foods”), whereas this particular item reflects a more passive form of Modeling. Furthermore, we could postulate that if caregivers practice healthy eating, the probability of healthy foods being available at home is high [64]. To elucidate, Melbye et al. [64] proposed that “healthy eating practices among parents might be more related to the availability of healthy foods in the home environment than to ‘active’ modeling of healthy eating” (p. 8).

In line with the results of Saltzman et al. [65], in the present study, the Involvement subscale included one item from the Teaching about Nutrition subscale (item 25, “I discuss with my child why it’s important to eat healthy foods”). In previous studies, items from feeding practices such as Involvement, Teaching about Nutrition, Encourage Balance and Variety, and Modeling also loaded together. Moreover, in some studies, items from these subscales were included in one new single factor named Healthy Eating Guidance [48,50,61,63,65]. This is expected because caregivers who use positive/responsive feeding practices do not use them in isolation but in combination with others [47,48,50,66].

The modified CFPQ subscales also showed adequate convergent and discriminant validity, which was demonstrated by the correlations between theoretically related constructs and an absence of correlations between unrelated constructs, respectively. In fact, the CFPQ Monitoring, Restriction for Weight Control, Restriction for Health, and Pressure subscales were strongly correlated with the corresponding CFQ subscales that measure the same constructs. Furthermore, distinct positive/responsive feeding practices such as Monitoring and Modeling were positively interrelated, and negative/non-responsive feeding practices such as Restriction, Emotion Regulation, and Pressure were also interrelated. Nonetheless, we found three apparently counterintuitive correlations. First, the CFPQ Environment subscale was negatively correlated with the CFQ Monitoring subscale. We believe it is possible that parents who keep less healthy foods or more unhealthy foods (e.g., salty snacks, candy, pastries) in the house feel the need to monitor their child’s intake of unhealthy foods. Second, the CFPQ Restriction for Health subscale was positively associated with the CFQ Monitoring subscale. This correlation was also found in previous studies [40,65]. As suggested by Saltzman et al. [65], caregivers who monitor their child’s food consumption could be more likely to guide them to eat healthy foods and thus may also restrict the child’s intake of unhealthy foods more. Lastly, the CFPQ Modeling subscale was positively correlated with the CFQ Restriction subscale. This correlation was also reported in Saltzman et al.’s [65] study, which could suggest parents who demonstrate healthy eating for their child may be more likely to also guide and restrict their child’s intake of food to limit the consumption of unhealthy foods.

This study has limitations that should be addressed. First, participants were recruited using convenience sampling procedures, and the large majority of the sample was highly educated and consisted of mothers of, particularly, preschool children, thereby limiting the generalization of the findings. Future research should test the CFPQ in more heterogeneous samples, as previous research has shown, for example, differences between mothers and fathers in the endorsement of feeding practices, with fathers reporting the use of more non-responsive feeding practices compared to mothers [67,68]. Similarly, parents with higher education levels are less likely to use non-responsive feeding practices [69,70,71]. Second, data are cross-sectional, limiting our ability to examine the stability of factor scores across time. Third, since the CFPQ is a self-report measure, participants’ responses could have been influenced by social desirability bias. Finally, other psychometric qualities should be assessed, like predictive validity with other measures of child eating behaviors and weight outcomes.

These limitations notwithstanding, given the lack of valid measures assessing parental feeding practices for the Portuguese population, the present study provides a relevant contribution by validating an instrument that covers a wide variety of feeding practices. Moreover, reducing the questionnaire from 49 to 29 items could decrease the response burden. Our results also add to the literature on parental feeding practices by supporting the role of cultural background and children’s age group in caregivers’ endorsements of feeding practices [23,33,69]. Indeed, as in previous validation studies, our study resulted in a modified version of the CFPQ. In this sense, the design and implementation of interventions targeting feeding practices to promote children’s development of healthy eating behaviors and prevent obesity must be culturally sensitive and appropriate to the child’s age. To make this possible, further large-scale studies that include culturally diverse samples are needed to facilitate cross-culture comparisons and understand cultural differences in parental feeding practices.

## Figures and Tables

**Table 1:** Subscales’ internal consistency reliabilities (α) for the 12-factor CFPQ model.

Subscale	α
Monitoring (4 items: 1, 2, 3, 4)	0.85
Modeling (3 items: 46, 47, 48)	0.77
Teaching Nutrition (1 item: 25)	
Balance and Variety (3 items: 24, 26, 38)	0.63
Environment (4 items: 14, 16R, 22, 37R)	0.66
Involvement (3 items: 15, 20, 32)	0.69
Emotion Regulation (3 items: 7, 8, 9)	0.82
Food Reward (3 items: 19, 23, 36)	0.59
Child Control (2 items: 5, 6)	0.46
Restriction Weight (6 items: 27, 29, 33, 34, 35, 41)	0.81
Restriction Health (3 items: 21, 28, 40)	0.64
Pressure (3 items: 30, 39, 49)	0.74

**Table 2:** Items, factor loadings, and Cronbach’s α for the 8-factor CFPQ model.

Factors and Items	Original Factors	Factor Loading
Monitoring (α = 0.85)		
1	Monitoring	0.81
2	Monitoring	0.91
3	Monitoring	0.64
4	Monitoring	0.73
Modeling (α = 0.77)		
46	Modeling	0.59
47	Modeling	0.93
48	Modeling	0.78
Environment (α = 0.59)		
14	Environment	0.58
44	Modeling	0.47
Involvement (α = 0.69)		
15	Involvement	0.61
20	Involvement	0.76
25	Teaching Nutrition	0.41
32	Involvement	0.60
Emotion Regulation (α = 0.82)		
7	Emotion Regulation	0.62
8	Emotion Regulation	0.88
9	Emotion Regulation	0.87
Restriction for Weight (α = 0.81)		
27	Restriction for Weight	0.70
29	Restriction for Weight	0.77
33	Restriction for Weight	0.68
34	Restriction for Weight	0.51
35	Restriction for Weight	0.53
41	Restriction for Weight	0.52
Restriction for Health (α = 0.63)		
21	Restriction for Health	0.69
28	Restriction for Health	0.59
43	Restriction for Health	0.45
Pressure (α = 0.73)		
17	Pressure	0.45
30	Pressure	0.65
39	Pressure	0.72
49	Pressure	0.70

**Table 3:** Associations between the 8-factor CFPQ model subscales and CFQ subscales.

		CFQ		
		Restriction	Pressure to Eat	Monitoring
CFPQ	Monitoring	0.02	−0.11	0.41 ***
	Modeling	0.25 ***	−0.04	0.24 ***
	Environment	−0.20 ***	−0.14 *	−0.14 *
	Involvement	−0.15 *	−0.13 *	0.03
	Emotion Regulation	0.19 **	0.11	0.00
	Restriction for Weight	0.31 ***	0.10	0.12
	Restriction for Health	0.65 ***	0.27 ***	0.22 ***
	Pressure	0.32 ***	0.77 ***	−0.02

* *p* < 0.05; ** *p* < 0.01; *** *p* < 0.001.

## Data Availability

Data are contained within the article.
